# Differential Regulation of Adhesion Complex Turnover by ROCK1 and ROCK2

**DOI:** 10.1371/journal.pone.0031423

**Published:** 2012-02-13

**Authors:** Frances E. Lock, Katie R. Ryan, Natalie S. Poulter, Maddy Parsons, Neil A. Hotchin

**Affiliations:** 1 School of Biosciences, University of Birmingham, Edgbaston, United Kingdom; 2 Randall Division of Cell and Molecular Biophysics, King's College London, London, United Kingdom; Institute of Developmental Biology and Cancer Research, France

## Abstract

**Background:**

ROCK1 and ROCK2 are serine/threonine kinases that function downstream of the small GTP-binding protein RhoA. Rho signalling via ROCK regulates a number of cellular functions including organisation of the actin cytoskeleton, cell adhesion and cell migration.

**Methodology/Principal Findings:**

In this study we use RNAi to specifically knockdown ROCK1 and ROCK2 and analyse their role in assembly of adhesion complexes in human epidermal keratinocytes. We observe that loss of ROCK1 inhibits signalling via focal adhesion kinase resulting in a failure of immature adhesion complexes to form mature stable focal adhesions. In contrast, loss of ROCK2 expression results in a significant reduction in adhesion complex turnover leading to formation of large, stable focal adhesions. Interestingly, loss of either ROCK1 or ROCK2 expression significantly impairs cell migration indicating both ROCK isoforms are required for normal keratinocyte migration.

**Conclusions:**

ROCK1 and ROCK2 have distinct and separate roles in adhesion complex assembly and turnover in human epidermal keratinocytes.

## Introduction

Signalling through Rho family GTPases plays a fundamental role in regulating cell interaction with extracellular matrix (ECM) via heterodimeric adhesion receptors known as integrins [1]. Integrins act as bidirectional signal transducers and are clustered into structures generically referred to as adhesion complexes [2]. Initially these originate as ‘focal complexes’ and form in response to signalling through Rac or Cdc42 [3]. Focal complexes are small adhesion structures which are either relatively rapidly turned over, or mature into much larger, longer-lived ‘focal adhesions’ [4]. The transition from focal complex to focal adhesion is, in part, a function of RhoA and its downstream effectors - ROCK1 and ROCK2 - which stimulate acto-myosin contractility and also mDia which can induce growth of focal complexes in a ROCK-independent manner [5], [6]. However, the exact roles played by ROCK1 and ROCK2 in regulating adhesion complex formation and function is yet to be elucidated. Although ROCK1 and ROCK2 share 92% amino acid sequence identity across their kinase domains, sequence identity drops to 65–70% across their PH domains, which may account for the observed differences in cellular localisation of the two isoforms [7], [8]. Both isoforms of ROCK play a role in regulating the acto-myosin cytoskeleton through phosphorylation, and inhibition, of the regulatory subunit of myosin light-chain phosphatase [9], [10]. In addition, ROCK1, but not ROCK2, can also phosphorylate, and activate, myosin light chain and both of these phosphorylation events serve to promote acto-myosin contractility [7].

Much is still to be learnt about the mechanism of adhesion complex assembly and maturation but the role of the non-receptor tyrosine kinase FAK is well established [11]. Adhesion to ECM results in activation of FAK which in turn facilitates recruitment of a large number of cytoskeletal and cytosolic proteins into focal complexes which in turn leads to cytoskeletal remodelling and the formation of the more mature focal adhesions [4], [11]. These large, elongated structures are associated with actin- and myosin-containing filament bundles (stress fibres) [12]. FAK also plays a key role in cell migration regulating assembly and disassembly of adhesion complexes at the leading edge of migrating cells [13].

Signalling through integrins is implicated in a wide variety of cellular events including cell cycle progression, cell survival, cell migration and differentiation. One example of this is the human epidermis, where terminal differentiation of epidermal keratinocytes is closely linked to integrin function [14]. Previous data from our laboratory linked activation of ROCK to the onset of terminal differentiation and more recently we have demonstrated distinct and opposing roles for ROCK1 and ROCK2 in the regulation of keratinocyte differentiation and adhesion to fibronectin [15], [16]. In this study we analyse the individual roles of ROCK1 and ROCK2 in adhesion complex assembly and identify distinct and separate roles for the two kinases.

## Results

### Focal adhesion formation is differentially regulated by ROCK1 and ROCK2

**Figure 1:**
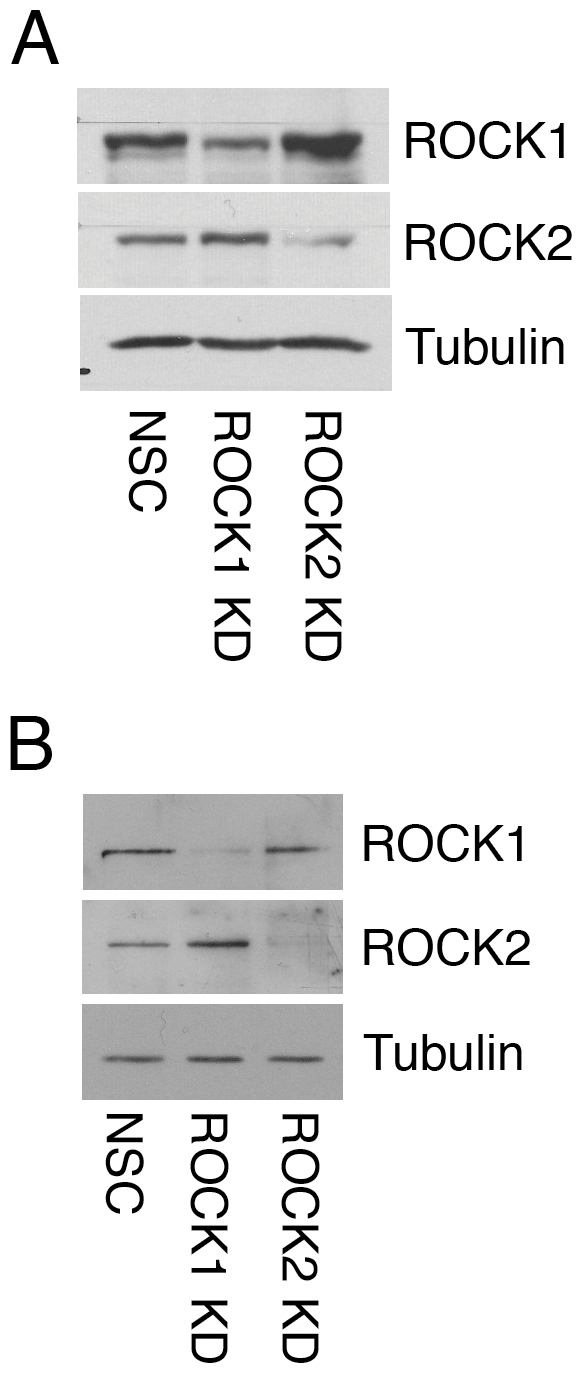
Stable and transient knock-down of ROCK1 and ROCK2 expression in two different keratinocyte cell lines. A: HaCaT keratinocytes stably expressing control (NSC), ROCK1 (ROCK1 KD) or ROCK2 (ROCK2 KD) shRNA vectors or B: SCC12f keratinocytes transiently transfected with NSC, ROCK1 or ROCK2 RNAi oligos were lysed and immunoblotted with antibodies against ROCK1, ROCK2 or tubulin as a loading control.

**Figure 2:**
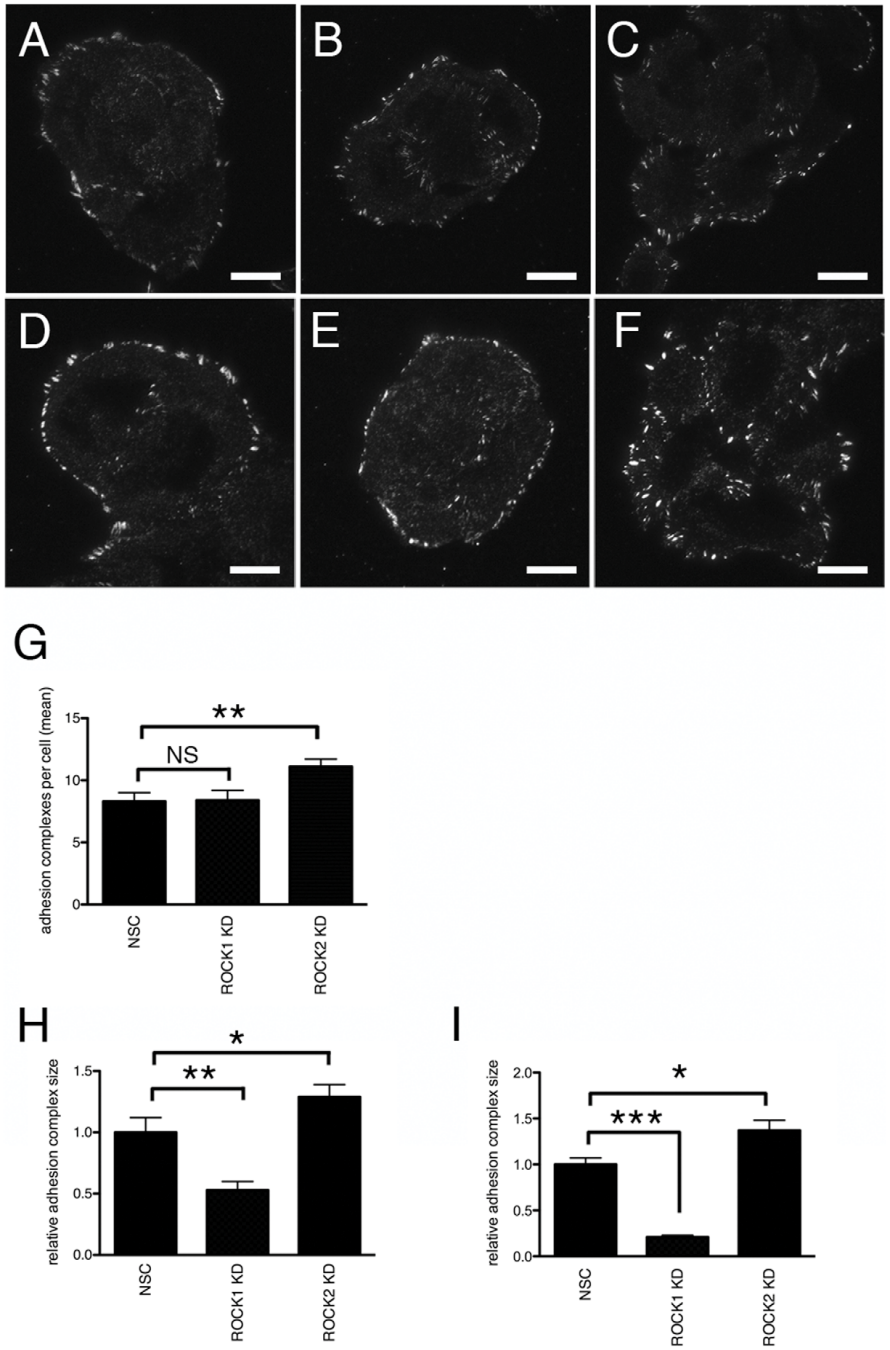
Adhesion complex formation in ROCK1- and ROCK2-depleted keratinocytes. HaCaT-NSC (A, D), HaCaT-ROCK1-KD (B, E) and HaCaT-ROCK2-KD (C, F) were cultured on glass coverslips and stained with antibodies against paxillin (A–C) or phospho-Y118 paxillin (D–F) and imaged using TIRF microscopy. Representative images from a minimum of 3 separate experiments are shown. Scale bar = 25 µm. The mean number of adhesion complexes (G) and mean area of adhesion complexes stained with anti-paxillin (H) or anti-phospho-Y118 paxillin (I) was calculated from 3 separate experiments (minimum 75 cells per experiment) and analysed using unpaired two-way Student's T-test (**p<0.01 *p<0.05).

In previously published work we described the RNAi-mediated knockdown of ROCK1 and ROCK2 in human keratinocytes. These knockdowns are isoform-specific, with no evidence for either isoform up-regulating expression or activity of the other isoform [16]. We also demonstrated that depletion of ROCK1 and ROCK2 expression has distinct effects on keratinocyte adhesion to fibronectin. Loss of ROCK1 expression resulted in decreased adhesion to fibronectin whereas depletion of ROCK2 resulted in increased adhesion [16]. We extended these studies to the analysis of adhesion complex formation and turnover in keratinocytes in which expression ROCK1 or ROCK2 was depleted (Figure 1). Paxillin is a multi-domain protein associated both with small, newly formed, ‘focal complexes’ and with more mature and larger ‘focal adhesions’ [17]. We used TIRF microscopy to analyse adhesion structures in these cells. In control cells, paxillin was localised to adhesion structures at the periphery of the cells (Figure 2A). In ROCK1-depleted cells we observed smaller adhesion structures (Figure 2B). This was in contrast to ROCK2-depleted cells where we observed an apparent increase both in numbers and in size of adhesion complexes (Figure 2C). ROCK1- and ROCK2-depleted cells were also stained with antibodies raised against phosphorylated paxillin (Y118). Tyrosine 118 of paxillin is a substrate for focal adhesion kinase (FAK) and Src and phosphorylation at Y118 is required for integrin-mediated cytoskeletal reorganization, FA turnover and maturation, and cell migration [18], [19]. In ROCK1 knockdown cells we observed a clear decrease in phospho-paxillin staining, whereas in ROCK2-depleted cells we observed increased staining (Figure 2D–F). Transfection of an alternative epidermal keratinocyte cell line SCC12f with ROCK1 and ROCK2 siRNA oligos resulted in similar changes in adhesion complex size (Figure S1). To analyse this further we used image analysis software to quantify numbers, and size, of paxillin-positive adhesion complexes in these cells. In ROCK2-depleted cells we observed a significant increase in both numbers and size of adhesion complexes (Figure 2G–I). In contrast, in ROCK1-depleted cells we did not observe any significant difference in numbers of adhesion complexes when compared to control cells (Figure 2G), but we did observe a significant decrease in complex size (Figure 2H–I). No difference in cell size was observed when ROCK1 and ROCK2 knockdown cells were compared to control cells (data not shown).

**Figure 3:**
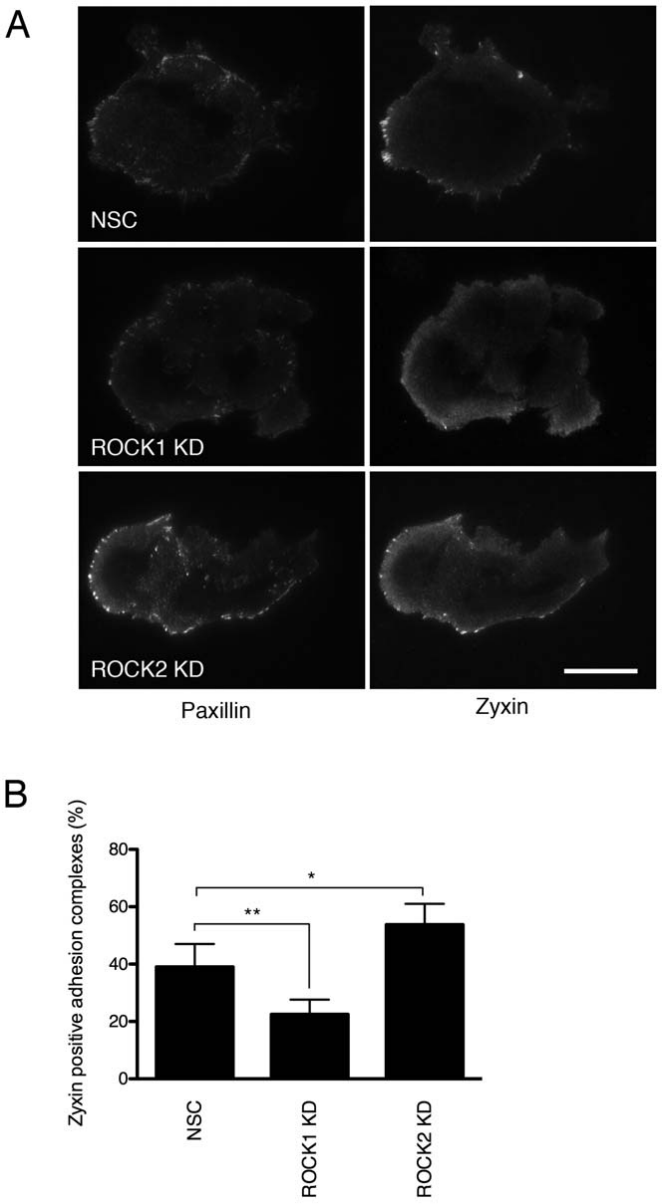
Localisation of zyxin to adhesion complexes in ROCK1- and ROCK2-depleted keratinocytes. A: HaCaT-NSC, HaCaT-ROCK1-KD and HaCaT-ROCK2-KD keratinocytes were cultured on glass coverslips and stained with an antibody against zyxin and imaged using TIRF microscopy. Representative images from a minimum of 3 separate experiments are shown. Scale bar = 25 µm B: The mean number of zyxin positive adhesion complexes was calculated as a function of the total number of paxillin-labelled complexes and analysed using an unpaired two-way Student's T-test (**p<0.01 *p<0.05).

Localisation of another adhesion marker, zyxin, was also assessed using TIRF microscopy. Zyxin is a marker of mature focal adhesions and is notably absent from smaller immature focal complexes [20]. In control NSC cells, zyxin was predominantly localised to focal adhesions at the periphery of the cell (Figure 3). In ROCK1-depleted cells, we observed relatively few zyxin-positive focal adhesions, suggesting that only immature focal complexes are present in the absence of ROCK1 (Figure 3). In contrast, following ROCK2 knockdown, we observed zyxin in large, mature focal adhesions at the periphery of the cell (Figure 3).

**Figure 4:**
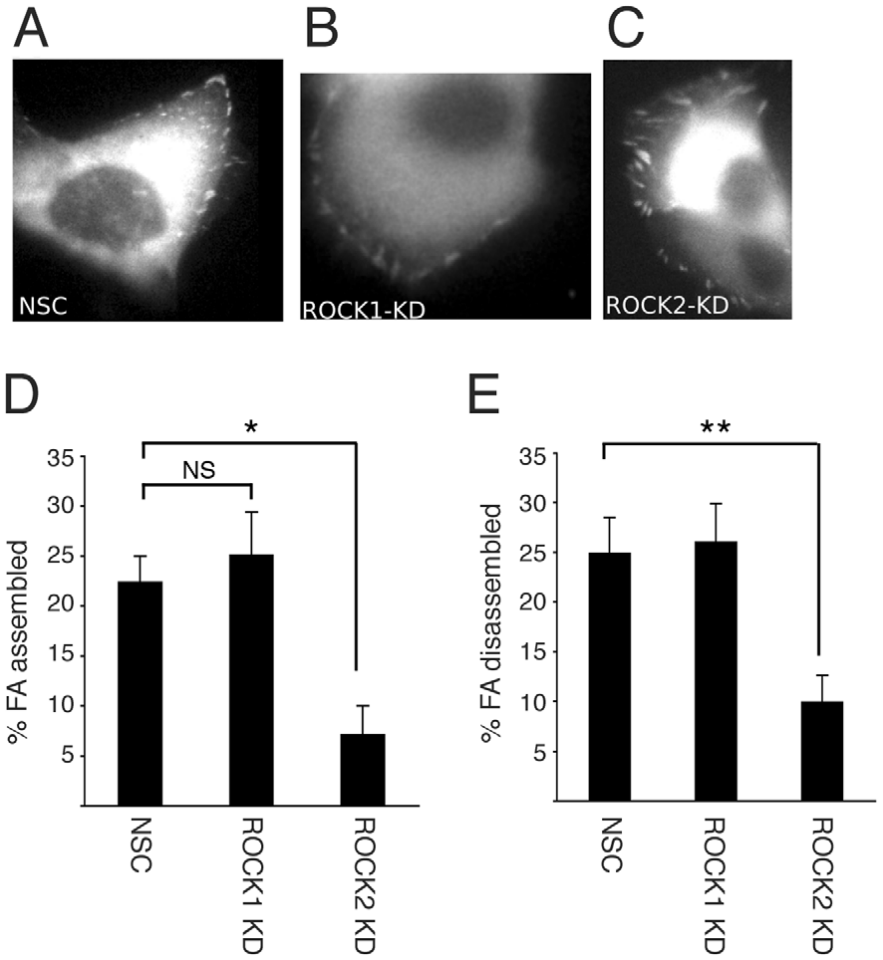
Adhesion complex turnover in ROCK1- and ROCK2-depleted keratinocytes. Focal adhesion size and dynamics were analysed in HaCaT-NSC, HaCaT-ROCK1-KD and HaCaT-ROCK2-KD cells transiently transfected with mRFP-paxillin. Live images of adhesion complexes were taken every 20 seconds for 20 minutes and representative images are shown (A–C). Rates of adhesion complex assembly (D) and disassembly (E) were calculated. A minimum of 12 cells per population was analysed in each experiment and the data are the mean and standard error from 3 separate experiments. Statistical analysis was performed using a Mann Whitney test (**p<0.01 *p<0.05). See Videos S1, S2 and S3 for representative movies).

The observation that zyxin was present in adhesion complexes in ROCK2- but not ROCK1-depleted cells suggested there might be differences in adhesion complex assembly and turnover. To address this, we used spinning disk fluorescent time-lapse microscopy to monitor recruitment and loss of red fluoresecent protein (RFP)-paxillin in adhesion complexes in ROCK1- and ROCK2-depleted cells (Figure 4). HaCaT-NSC, HaCaT-ROCK1-KD and HaCaT-ROCK2-KD cells expressing RFP-paxillin were plated on coverslips coated with fibronectin and focal adhesions imaged. Stills from each movie are shown in Figure 4A–C (see Videos S1, S2 and S3 for representative movies). Control HaCaT-NSC cells contained adhesion complexes that assembled (Figure 4D) and disassembled (Figure 4E) during the 20 min duration of the assay. Interestingly, despite possessing significantly smaller adhesion complexes (Figure 2H) ROCK1-depleted cells exhibited adhesion complex dynamics similar to those of controls cells (Figure 4D,E). In contrast, the large focal adhesions present in HaCaT-ROCK2-KD cells showed very little recruitment or loss of RFP-paxillin (Figure 4D,E).

### ROCK1 expression is required for FAK phosphorylation

A key component of both focal complexes and focal adhesions is focal adhesion kinase (FAK). FAK is a non-receptor tyrosine kinase that is activated and localised to adhesion complexes upon cell adhesion to ECM and is involved in adhesion complex remodelling during cell migration [11]. Much work has focussed on the numerous tyrosine residues present on FAK that are differentially phosphorylated in response to various agonists and are implicated in different signalling pathways [11]. FAK also has four serine residues in the C-terminal tail - S722, S732, S843, and S910 - the functions of which are still largely unknown. Recent work in endothelial cells has demonstrated that over-expression of a constitutively active form of ROCK results in phosphorylation of FAK on S732 and that this phosphorylation event is essential for phosphorylation of FAK Y407 and VEGF-stimulated cell movement [21], [22]. Furthermore, expression of a non-phosphorylatable mutant of FAK (S732A) inhibited recruitment of vinculin to focal adhesions [21]. We confirmed the importance of S732 phosphorylation for adhesion complex assembly by transfecting SCC12f keratinocytes with a GFP-IRES-FAK cDNA expression construct in which serine 732 is mutated to alanine (FAK^S732A^). When cells expressing FAK^S732A^ were fixed and stained for zyxin as a marker for mature focal adhesions we observed greatly reduced zyxin staining in cells expressing FAK^S732A^ when compared to control cells (Figure S2).

**Figure 5:**
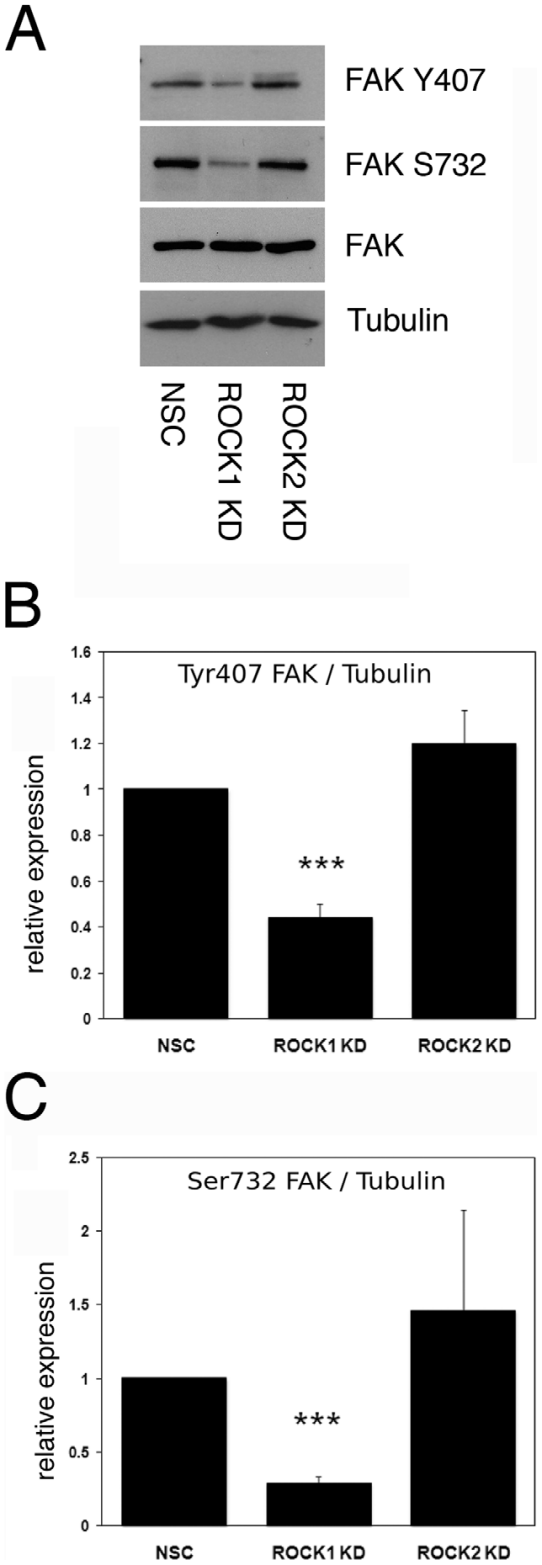
Reduced FAK phosphorylation in ROCK1-depleted keratinocytes. A: HaCaT-NSC, HaCaT-ROCK1-KD or HaCaT-ROCK2-KD cells were lysed and immunoblotted to analyse phosphorylation of FAK residues serine^732^ (FAK S732) and tyrosine^407^ (FAK Y407). Total FAK expression was also analysed and tubulin expression were assessed as a loading control. Densitometry was used to quantitate phosphorylation of FAK tyrosine^407^ (B) and serine^732^(C). Data shown are the mean and standard error from three separate experiments and statistical analysis was carried out using a Mann-Whitney test (*** p<0.001).

HaCaT-NSC, HaCaT-ROCK1-KD and HaCaT-ROCK2-KD cell lysates were immunoblotted to assess changes in phosphorylation of FAK S732. A significant decrease in S732 phosphorylation was observed in ROCK1-depleted cells, when compared to control NSC cells (Figure 5A). Consistent with FAK S732 being a substrate for ROCK1 but not ROCK2, we observed no significant change in S732 phosphorylation in ROCK2 knockdown cells (Figure 5A, B). Phosphorylation of FAK on S732 is a requirement for FAK Y407 phosphorylation, which in turn is required for recruitment of vinculin and subsequent adhesion complex maturation [21]. Consistent with our finding that loss of ROCK1 results in decreased S732 phosphorylation, we also observed a significant decrease in Y407 phosphorylation in HaCaT-ROCK1-KD cells when compared to control cells (Figure 5A, C). No significant change in Y407 phosphorylation was observed in ROCK2-depleted cells (Figure 5A, C).

### Depletion of ROCK1 or ROCK2 inhibits scratch wound closure

**Figure 6:**
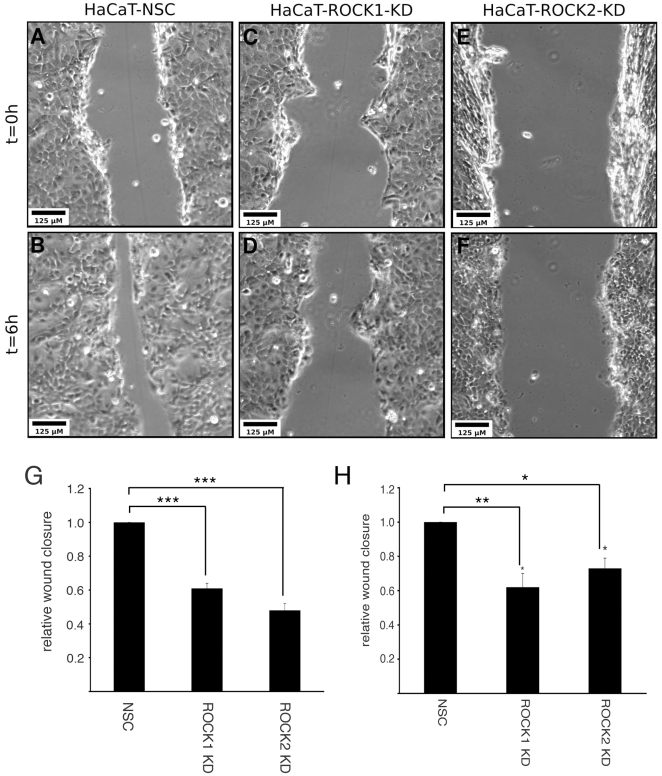
Loss of either ROCK1 or ROCK2 expression results in impaired cell migration. Confluent HaCaT-NSC (A,B), HaCaT-ROCK1-KD (C,D) and HaCaT-ROCK2-KD (E,F) monolayers were scratched with a pipette tip. Phase contrast images were taken immediately and 6 hours after wounding and representative phase-contrast images are shown (A–F). Relative wound closure was calculated for HaCaT-ROCK1-KD and HaCaT-ROCK2-KD cells (G) as well as SCC12f keratinocytes transiently transfected with siRNA directed against ROCK1 and ROCK2 (H). The means and standard errors of 3 separate experiments are shown and statistical significance calculated using Mann-Whitney test (*** p<0.001, * p<0.05).

Having established that ROCK1 and ROCK2 have distinct roles in the organisation of adhesion complexes we used a scratch-wound assay to analyse the ability of ROCK1- and ROCK2-depleted keratinocytes to migrate into the ‘wounded’ area. HaCaT cells stably transfected with shRNA targeting ROCK1 and ROCK2 or a non-silencing control (NSC) were plated onto fibronectin coated dishes, wounded with a pipette tip and wound closure analysed. We observed inhibition in wound closure in both ROCK1-depleted HaCaT cells (Figure 6C, D) and in ROCK2-depleted cells (Figure 6E, F) when compared to NSC-transfected cells (Figure 6A, B). Statistical analysis of multiple experiments revealed significant inhibition (p<0.001) of wound closure in both ROCK1- and ROCK2-depleted cells (Figure 6G). Transient transfection of an alternative keratinocyte cell line – SCC12f - with siRNA oligos directed against different ROCK1 and ROCK2 sequences revealed a similar inhibition of cell migration in both ROCK1- and ROCK2- depleted cells (Figure 6H).

## Discussion

Despite the biochemical evidence that ROCK1 and ROCK2 do not always share common downstream targets, most analysis of ROCK function has relied on either over-expression of one ROCK isoform or use of small molecule inhibitors, such as Y-27632, which inhibit both ROCK1 and ROCK2 [23]. Both approaches have significant drawbacks and neither discriminate ROCK1 and ROCK2 function. In vivo data show that, despite their high sequence identity, ROCK1 or ROCK2 expression cannot compensate for loss of the other isoform during murine embryonic development [24], [25]. More recently, in vitro studies using isoform-specific RNA interference demonstrated that ROCK1 and ROCK2 have distinct, and sometimes opposing, roles in fibroblasts [8], [26]. Consistent with this, data from our laboratory demonstrated that ROCK 1 and ROCK2 differentially regulate adhesion to fibronectin and terminal differentiation in human epidermal keratinocytes [16]. In this study we have demonstrated that ROCK1 and ROCK2 differentially regulate assembly and maturation of adhesion complexes.

### ROCK1 regulates FAK phosphorylation and adhesion complex maturation in keratinocytes

Integrin-based adhesion complexes are dynamic structures that must assemble, disassemble or mature at the extending leading edge and disassemble at the retracting cell rear for efficient cell migration to occur [4]. FAK is a key regulator of adhesion complex assembly and disassembly and we observed that depletion of ROCK1, but not ROCK2, in keratinocytes resulted in a significant decrease in FAK phosphorylation at both Tyr407 and Ser732. Regulation of FAK activity via Rho/ROCK signalling has been reported in a number of different cell types [21], [22], [27], [28]. Using an in vitro kinase assay, constitutively active ROCK1 has been shown to phosphorylate a fragment of FAK at Ser732, an event that is required to trigger the formation of focal adhesions in endothelial cells following VEGF treatment [22]. Maximal activity of FAK requires phosphorylation at Tyr407. Le Boeuef et al (2006) proposed a mechanism whereby ROCK1 phosphorylates FAK at Ser732 and that this phosphorylation is required for subsequent phosphorylation of Tyr407, possibly by Pyk2 [22]. We propose that the failure of focal complexes to mature into focal adhesions in ROCK1-depleted cells is a consequence of ROCK1 not being available to phosphorylate FAK at Ser732.

Phosphorylation of FAK at Ser732 has been linked to increased neuronal cell migration, and over-expression of the non-phosphorylatable FAK Ser732Ala mutant impairs cell movement in vitro and neuronal migration in vivo [29], [30]. In addition, in endothelial cells depletion of ROCK1 results in loss of Ser732 FAK phosphorylation and inhibition of endothelial cell migration through a Boyden chamber [22]. This strongly suggests that inhibition of keratinocyte migration following ROCK1 depletion is a consequence of decreased Ser732 FAK phosphorylation and subsequent reduction of Tyr407 FAK phosphorylation. This would place ROCK upstream of FAK. However, it is worth noting that there are several reports that place Rho signalling downstream of FAK [31], [32]. For example, FAK-dependent regulation of both p190RhoGAP and p190RhoGEF activity has been reported [33], [34]. This raises the possibility of both positive and negative feedback loops whereby ROCK1 activates FAK, and once activated, FAK positively or negatively regulates Rho activity via GEF or GAPs [35].

### ROCK signalling in adhesion complex formation and turnover

Actin stress fibres play an important role in the formation of focal adhesions. Acto-myosin-driven forces are necessary for the recruitment of integrin-associated molecules to the cytoplasmic tails of integrins and the subsequent maturation of focal complexes into focal adhesions and adhesion complex size has been found to be proportional to the force applied to it by the cell [36]. Treatment of cells with Y-27632 to inhibit ROCK1 and ROCK2 activity results in the accumulation of immature adhesion complexes and recent data points towards focal adhesions being highly mechano-sensitive structures [20], [37], [38]. Our data are consistent with a role for ROCK1 rather than ROCK2 in the maturation of focal complexes into focal adhesions. The observation that loss of ROCK2 expression results in reduced focal adhesion turnover is also consistent with previous data in which shRNA knockdown of ROCK2 resulted in adhesion complexes with significantly reduced fibroblast motility [13]. Interestingly, the same study also reported that shRNA-mediated depletion of ROCK2 resulted in decreased adhesion complex mobility as determined by TIRF [13]. Here, we demonstrate that whilst depletion of ROCK2 in keratinocytes resulted in smaller, less mature adhesion complexes, the rate of assembly and disassembly was unaffected.

### Both ROCK1 and ROCK2 are required for keratinocyte migration

Reorganisation of the actin cytoskeleton is required for cell migration and plays an essential role in the formation of leading edge filopodia and lamellipodia, both of which adhere to ECM via focal complexes and focal adhesions. During cell migration, the maturation of focal complexes into mature focal adhesions at the leading edge requires the tension provided by acto-myosin-driven contractility, which in turn provides the tensional forces required for motility [12]. At the rear or trailing edge of the cell, mature focal adhesions must be disassembled to release the cell from its substratum and allow forward movement. Hence, continuous assembly and disassembly of focal adhesions are necessary for continued cell movement and there is evidence for both ROCK1 and ROCK2 being required for fibroblast migration [4], [13], [39]. Thus, the decreased ‘wound closure’ observed in ROCK1-depleted keratinocytes is likely to be a consequence of focal complexes failing to mature into focal adhesions, thus depriving the cell of the acto-myosin contractile forces required for movement. In ROCK2-depleted keratinocytes focal adhesions are very stable and have much lower turnover rates compared to control cells, which is consistent with a previous report in which knockdown of ROCK2 inhibited LPA-stimulated trailing edge retraction in fibroblasts [13]. A recent paper analysing ROCK1 and ROCK2 function in PC3 prostate cancer cells reported that knockdown of either isoform had no significant effect on migration speed, but that knockdown of ROCK2 (but not ROCK1) resulted in decreased persistence in a chemotaxis assay, indicating a role for ROCK2 in directional migration in PC3 cells [40]. The differences between those findings and those reported here may reflect differences in motility; PC3 cells migrate as individual cells rather than confluent cell sheets as is seen in keratinocytes. Alternatively, cell-type specific differences in ROCK function may explain this apparent discrepancy. For example, we have observed previously that activation of ROCK results in cell cycle arrest in keratinocytes, whereas in fibroblasts activation of ROCK is required for cell cycle progression [15], [41].

## Materials and Methods

### Cell Culture

HaCaT keratinocytes and SCC12f keratinocytes were cultured using standard techniques as described elsewhere [16], [42]–[44]. Cell culture medium and reagents were purchased from Invitrogen. SCC12f keratinocytes were transiently transfected with pEGFP-C1 or pEGFP-C1-IRES-S732A-FAK (a gift from Li-Huei Tsai, Stanley Center for Psychiatric Research, Broad Institute of MIT and Harvard University, USA) using Lipofectamine 2000 reagent (Invitrogen) according to manufacturers instructions.

### RNAi

HaCaT keratinocytes were stably transfected with GFP-IRES-shRNAmir constructs which specifically target either ROCK1, ROCK2 or a non-silencing control nonsense mRNA sequence (NSC) to generate polyclonal HaCaT-ROCK1-KD, HaCaT-ROCK2-KD and HaCaT-NSC cell lines respectively. The establishment and analysis of ROCK-dependent phosphorylation in these cell lines is described elsewhere [16]. Plasmids were purchased from Open Biosystems UK as follows: ROCK1 (RHS4186-97556976); ROCK2 (RHS4430-98854581); NSC (RHS4346). In some experiments SCC12f were transiently transfected with siRNA oligos targeting ROCK1 (J-003536-06-0020, J-003536-07-0020, Thermo Scientific, USA), ROCK2 (S102223746, S102223753, QIAGEN, UK) or NSC (S103650325, QIAGEN, UK) as described elsewhere [16]. In all cases isoform-specific decreases in ROCK1 and ROCK2 expression were observed.

### Antibodies

Primary antibodies used were tubulin (T6199, Sigma, MO, USA); paxillin (Cell signalling Technology, Inc., MA, USA); Y118-paxillin (Cell signalling Technology, Inc., MA, USA); Zyxin (ZOL301, Abcam, Cambridge, UK; Ser732 FAK (Abcam, Cambridge, UK); Tyr407 FAK (SantaCruz, USA); Phalloidin-Alexa594 (Sigma, MO, USA); FAK (BD Transduction Laboratories, Oxford, UK); Y-397-FAK (BD Transduction Laboratories, Oxford, UK). Secondary antibodies were purchased from Jackson Immunoresearch (West Grove, PA, USA).

### Immunocytochemistry

HaCaT or SCC12f keratinocytes cultured on acid-washed glass coverslips, were fixed with 4% paraformaldehyde in PBS, permeabilised with 0.2% Triton-X-100 and stained with the appropriate primary and secondary antibodies as described previously [16]. For epifluoresence, cells were visualized using a Leica DMRB microscope equipped with a Hamamatsu ORCA camera, and images were captured and processed using OpenLab software (Improvision). For each immunostaining, the same exposure time was used to capture images.

### Total internal reflection fluorescence (TIRF) microscopy image acquisition

TIRF images were acquired on a Nikon TIRF system. Laser illumination was achieved through the microscope objective (CFI TIRF Apo 60x NA 1.49, Nikon) on a Nikon EclipseTi inverted microscope. The 488 nm line of an Argon-Ion laser was used to image cells labelled with an Alexa-488 secondary antibody. Images were captured on a 12-bit CCD camera (Ixon 1 M EMCCD) using Nikon NIS Elements software with exposure times of 100–300 ms. Image analysis and manipulation was carried out in Nikon NIS-Elements AR (version 3.2).

### Analysis of adhesion complexes

To analyse adhesion complex turnover cells were transfected with mRFP-paxillin as previously described [45]. 36 hours later cells were plated onto glass-bottom dishes coated with FN (10 µg ml^−1^). After 2 hours of adhesion, cells were imaged using a CARV spinning disk head attached to a Leica DMIRB wide field microscope. All images were acquired using a 40×/1.3 oil immersion objective at 37°C. Images were acquired using separate Cy3 excitation and emission filter sets (Ludl) controlled by automatic excitation and emission wheels (Ludl) on an Orca ER CCD camera (Hammamatsu Inc). Acquisition was performed using Andor IQ software (Andor Bioimaging). Frames were acquired every 20 seconds over 20 minutes. Analysis of focal adhesion disassembly kinetics was performed in NIH ImageJ (http://rsb.info.nih.gov/ij/index.html). A background correction mask was applied to all frames within a single movie to highlight the RFP-positive structures. Manual tracking of the disappearance or appearance of RFP-paxillin-positive adhesions was performed through the entire time-lapse series. The percentage of all adhesions that dissolved or appeared over time was calculated by tracking 12 cells in each population in 3 independent experiments. The mean time taken for adhesions to disappear in each cell line was calculated from measuring the time to dissolution for a number of adhesions from multiple cells. For each cell line, measurements were taken from 12–15 adhesions from 5 cells from 3 independent experiments. To analyse adhesion complex size and number, HaCaT cells were cultured on glass coverslips, stained using anti-paxillin antibodies, and epifluorescence images were captured. Images were analysed and the size and number of paxillin-stained FA per cell were quantified using ImageJ. At least 75 cells over 3 separate experiments were analysed.

### Scratch wound healing studies

Glass-bottomed culture dishes (MatTek Corporation, MA, USA) were pre-coated with 25 µg ml^−1^ fibronectin (Sigma) at 4°C overnight, washed in PBS, blocked with 10 mg ml^−1^ BSA in PBS at 37°C for 3 hours, then washed with PBS. HaCaT-KD cells or SCC12f cells transiently transfected with siRNA oligos were plated and allowed to recover for 24 hours at 37°C, 5% CO_2_ in normal culture media. Two hours prior to wounding, cells were treated with 4 ug ml^−1^ mitomycin C to inhibit proliferation. Confluent monolayers were wounded with a sterile pipette tip and culture media replaced with cell imaging media (CIM) (10 µM HEPES-HBSS pH7.4 with 5% FBS). Cells were imaged immediately and at 6 hrs (HaCaT) or 1 hr (SCC12f) post-wounding at the same position using a Nikon TE300 epifluorescence microscope (Amstelveen, The Netherlands). Acquisition and imaging software used was OpenLab 5.0 (Improvision, Coventry, UK). The extent of wound healing was defined as the remaining wound area over the original wound area, expressed as fold change compared to the RNAi non-silencing control cells.

### SDS-PAGE and Western Blotting

Protein lysates were prepared in 3× Laemmli buffer, separated by SDS-PAGE, and immunoblotted as described elsewhere [16]. All experiments were performed on three separate occasions, with representative blots shown. Densitometry analysis was carried out using NIH ImageJ.

## Supporting Information

Figure S1 **Adhesion complex formation in ROCK1- and ROCK2-depleted keratinocytes.** SCC12f keratinocytes were transiently transfected with siRNA oligos against ROCK1 (C,D) or ROCK2 (E,F). As a control cells were transfected with non-silencing oligos (A,B). To visualise adhesion complexes cells were cultured on glass coverslips for 48 hours and stained with antibodies against paxillin (A,C,E) or FAK (B,D,F). Representative images from a minimum of 3 separate experiments are shown.
(TIF)

Figure S2 **Expression of S732A FAK inhibits focal adhesion formation.** Keratinocytes were cultured on glass coverslips and transiently transfected with pEGFP-C1 (A,B) or pEGFP-C1-IRES-S732A-FAK (C,D). After 24 hours, cells were stained with an antibody against zyxin (A,C). To confirm plasmid expression EGFP expression was monitored (B,D). Representative images from 3 separate experiments are shown.
(TIF)

Video S1 **Video showing representative HaCaT-NSC cells transfected with mRFP-paxillin and imaged for 20 minutes.** Frames were acquired every 20 seconds.
(AVI)

Video S2 **Video showing representative HaCaT-ROCK1-KD cells transfected with mRFP-paxillin and imaged for 20 minutes.** Frames were acquired every 20 seconds.
(AVI)

Video S3 **Video showing representative HaCaT-ROCK2-KD cells transfected with mRFP-paxillin and imaged for 20 minutes.** Frames were acquired every 20 seconds.
(AVI)
