# Supplementary figures and images for: Differential Regulation of Adhesion Complex Turnover by ROCK1 and ROCK2
Source: PLoS One. 2012 Feb 13;7(2):e31423. doi: 10.1371/journal.pone.0031423 (PMC3278444; doi:10.1371/journal.pone.0031423)

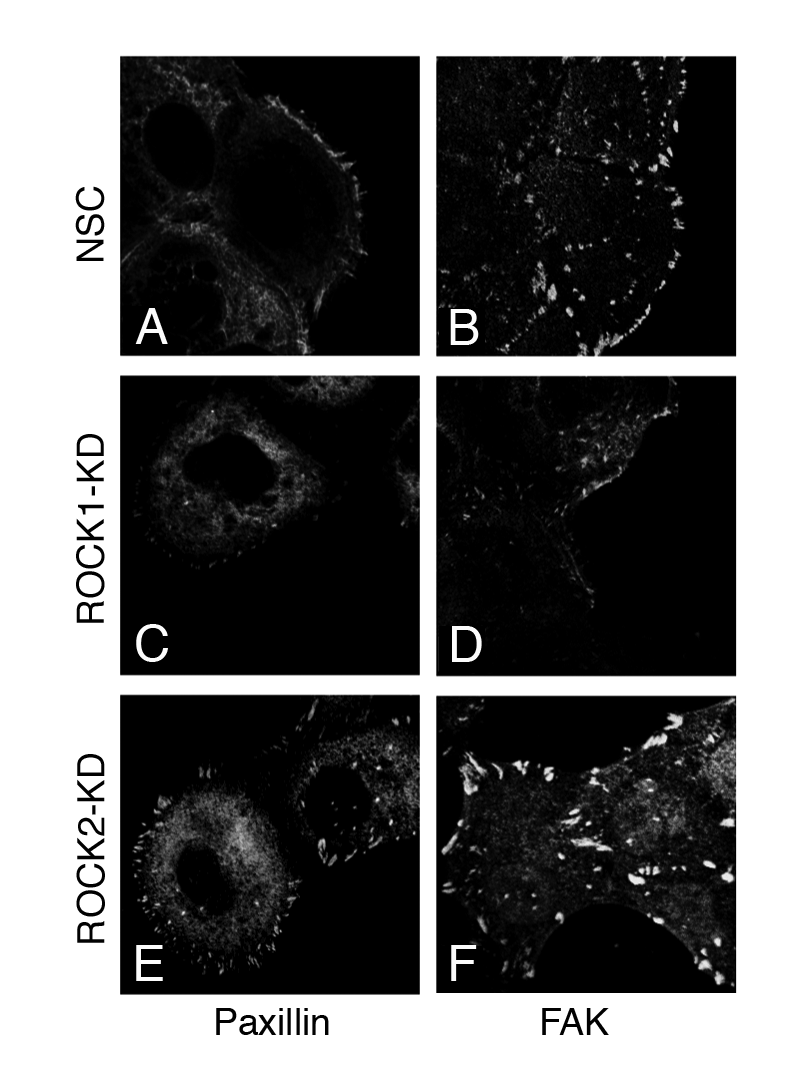

Supplement: Figure S1 — Adhesion complex formation in ROCK1- and ROCK2-depleted keratinocytes. SCC12f keratinocytes were transiently transfected with siRNA oligos against ROCK1 (C,D) or ROCK2 (E,F). As a control cells were transfected with non-silencing oligos (A,B). To visualise adhesion complexes cells were cultured on glass coverslips for 48 hours and stained with antibodies against paxillin (A,C,E) or FAK (B,D,F). Representative images from a minimum of 3 separate experiments are shown. (TIF) [file pone.0031423.s001.tif]

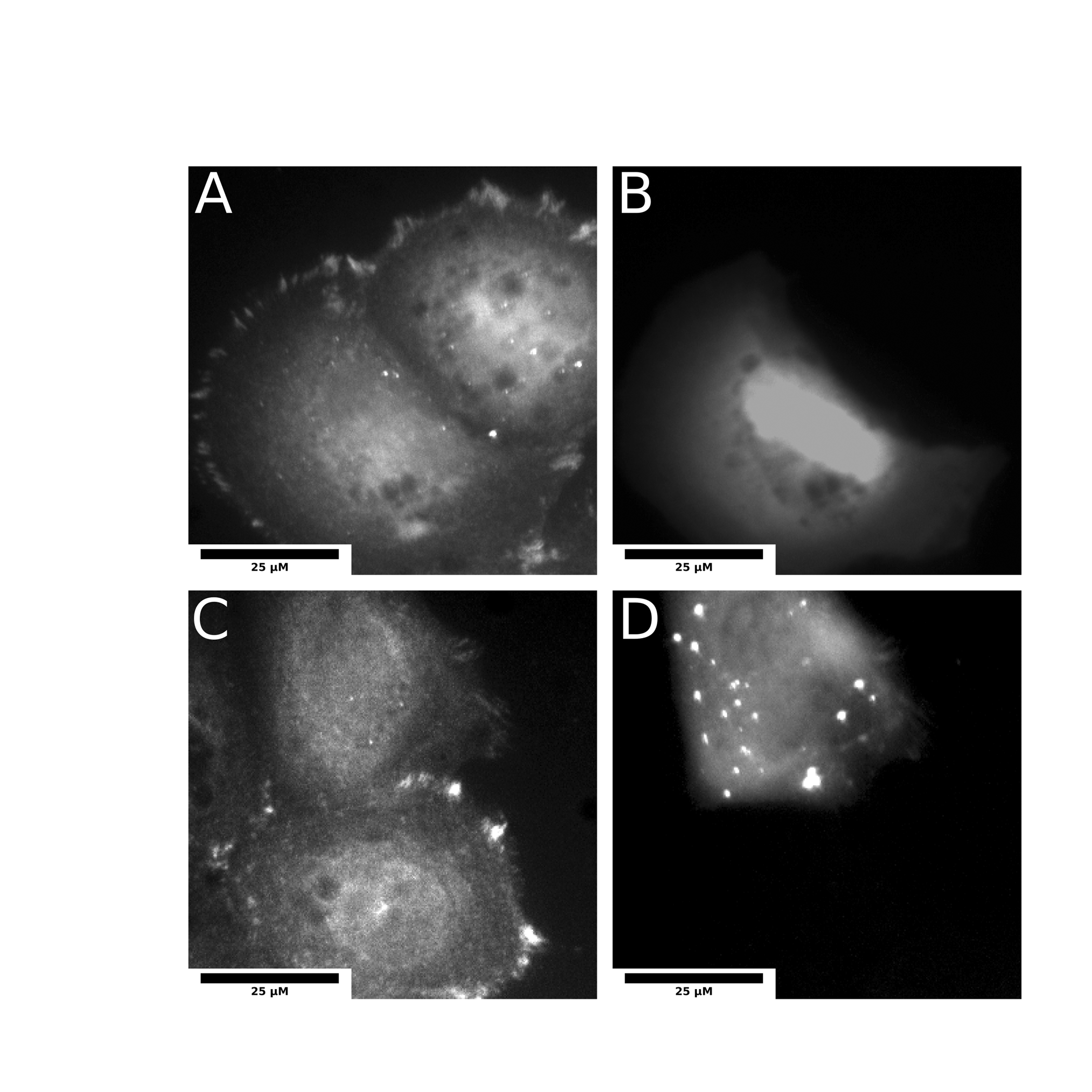

Supplement: Figure S2 — Expression of S732A FAK inhibits focal adhesion formation. Keratinocytes were cultured on glass coverslips and transiently transfected with pEGFP-C1 (A,B) or pEGFP-C1-IRES-S732A-FAK (C,D). After 24 hours, cells were stained with an antibody against zyxin (A,C). To confirm plasmid expression EGFP expression was monitored (B,D). Representative images from 3 separate experiments are shown. (TIF) [file pone.0031423.s002.tif]
